# The expression of p‐p62 and nuclear Nrf2 in esophageal squamous cell carcinoma and association with radioresistance

**DOI:** 10.1111/1759-7714.13252

**Published:** 2019-11-21

**Authors:** Zhe Wang, Jingze Zhang, Minghuan Li, Li Kong, Jinming Yu

**Affiliations:** ^1^ Department of Radiation Oncology, Shandong Cancer Hospital and Institute Shandong First Medical University and Shandong Academy of Medical Sciences Jinan China

**Keywords:** Concurrent chemoradiotherapy, long‐term survival, nuclear Nrf2, phosphorylated p62, treatment response

## Abstract

**Background:**

The roles of p62‐Keap1‐Nrf2 pathway in the radioresistance of esophageal squamous cell carcinoma (ESCC) have not yet been revealed. This study aimed to clarify the expression and correlation of p‐p62 and nuclear Nrf2 and their association with radioresistance in ESCC.

**Methods:**

This study included 164 cases of inoperable locally advanced ESCC. All patients received concurrent chemoradiotherapy (CCRT). Immunohistochemical staining was used to detect the expression of p‐p62 and nuclear Nrf2. The results were analyzed independently by two pathologists.

**Results:**

There was no significant relationship between p‐p62 or nuclear Nrf2 and patients' clinical characteristics. Compared to patients with low expression of p‐p62, patients with high expression of p‐p62 showed lower objective response rate (ORR). Similarly, patients with high expression of nuclear Nrf2 exhibited lower ORR compared to those with low expression of nuclear Nrf2. The expression of p‐p62 was positively correlated with that of nuclear Nrf2. Moreover, the correlation coefficient between them was higher among patients showing no response to CCRT. Univariate analysis revealed that higher expression of p‐p62 or nuclear Nrf2 was significantly associated with poorer PFS and OS. Multivariate analysis indicated that the expression of nuclear Nrf2 and treatment response were independent prognostic factors for PFS. Sex, treatment response, expression of p‐p62 and nuclear Nrf2 were independent prognostic factors for OS.

**Conclusion:**

Higher expression of p‐p62 and nuclear Nrf2 are associated with lower ORR as well as poorer prognosis, which indicates that p62‐Keap1‐Nrf2 pathway might play an essential role in the radioresistance of ESCC.

**Key points:**

- The expression of p‐p62 and nuclear Nrf2 in ESCC show a significant relationship with patients' responses to CCRT and influence the prognosis of ESCC.
- p62‐Keap1‐Nrf2 pathway might be a new target for radiosensitization in ESCC.

## Introduction

Esophageal cancer is one of the malignancies with the highest incidence and mortality rates worldwide. In China, esophageal squamous cell carcinoma (ESCC) is the most common pathological type. Because the symptoms of early ESCC lack specificities, most patients are in locally advanced or metastatic stage when they are initially diagnosed. Concurrent chemoradiotherapy (CCRT) is the standard therapy for inoperable locally advanced ESCC. Although CCRT shows a considerable response rate,^1^ most patients suffer recurrence or progression within three years,^1^, ^2^, ^3^ which implies that the molecular mechanisms underlying radioresistance of ESCC are crucial. Targeted therapy to increase the radiosensitivity of ESCC might be a breakthrough point for the treatment of ESCC. Many studies have demonstrated that reactive oxygen species (ROS) plays a critical role in anti‐tumor effects of radiotherapy by oxidizing DNA, protein, lipid etc.^4^, ^5^, ^6^, ^7^, ^8^, ^9^, ^10^, ^11^ Recent studies have revealed that the aberrant clearance of ROS is one of the mechanisms of radioresistance of ESCC.^12^, ^13^, ^14^

**Figure 1:**
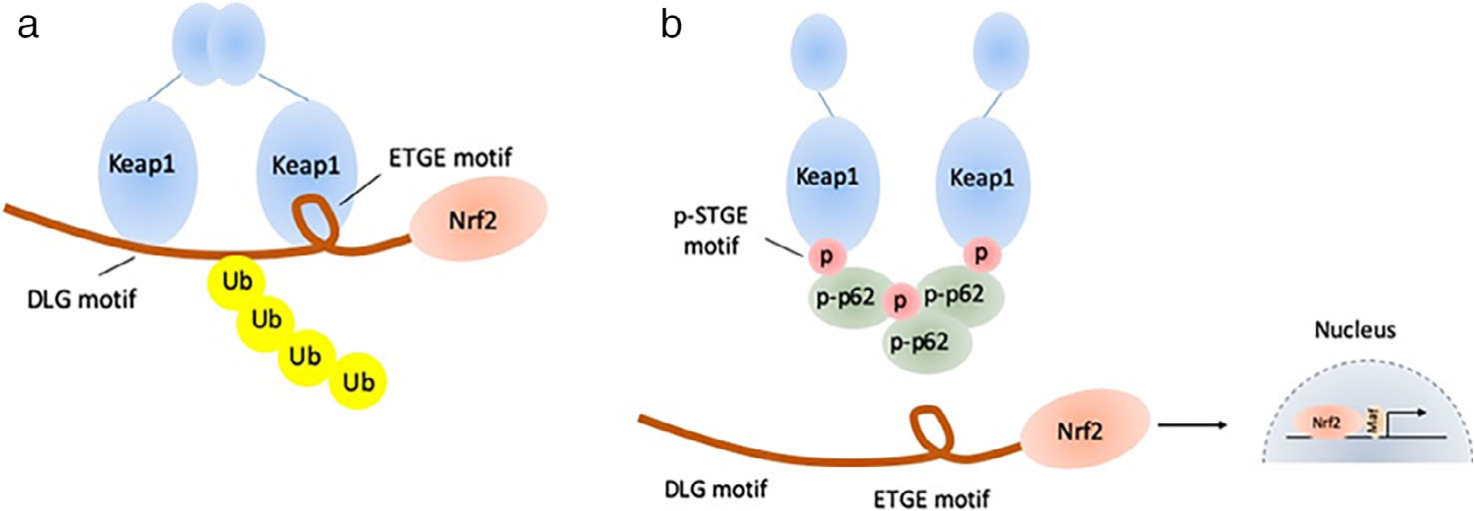
The illustration of p62‐Keap1‐Nrf2 pathway. (**a**) Under normal conditions, Keap1 combines with Nrf2 in the cytoplasm, which leads to the degradation of Nrf2 by ubiquitination. (**b**) Phosphorylated p62 has a similar domain with Nrf2 and shows a significantly higher affinity with Keap1. Phosphorylated p62 competitively combines with Keap1, which induces the release and activation of Nrf2. The activated Nrf2 promotes the transcription of multiple genes.

Kelch‐like ECH‐associated protein 1 (Keap1)‐ nuclear factor erythroid 2 related factor 2 (Nrf2)‐antioxidant response element (ARE) pathway is one of the important signal pathways regulating cellular ROS. Under normal condition, Keap1 combines with Nrf2 in the cytoplasm, which leads to the degradation of Nrf2. However, the conformation of Keap1‐Nrf2 complex changes under oxidative or ionized stress. The released Nrf2 enters into the nucleus and transcribes multiple antioxidative and detoxifying genes to protect the cells from oxidative damage.^15^, ^16^, ^17^, ^18^ The excessive activation of Keap1‐Nrf2 pathway is common in tumor cells and p62, a kind of autophagy adaptor, has been reported to be involved in this progression. Phosphorylated p62 (p‐p62) has a similar domain with Nrf2 and shows significantly higher affinity with Keap1. During selective autophagy, mTORC1 phosphorylates p62 at Ser349, and p‐p62 competitively combines with Keap1, which induces the release and activation of Nrf2.^19^ In addition, the activated Nrf2 promotes the transcription of p62 to form a positive loop.^20^, ^21^ The p62‐Keap1‐Nrf2 pathway is presented in Fig 1.

Several studies have demonstrated that the p62‐Keap1‐Nrf2 pathway is not only involved in the progression of oncogenesis^21^, ^22^ but also mediates anticancer agent tolerance.^23^, ^24^ Although several studies have proved that constant activation of Nrf2 is involved in the radioresistance in multiple cancers,^25^, ^26^, ^27^, ^28^, ^29^, ^30^, ^31^ the role of p62‐Keap1‐Nrf2 pathway is still unclear, especially in ESCC. The aim of this study was to clarify the expression of p‐p62 and nuclear Nrf2 in ESCC and their association with radioresistance.

## Methods

### Patients

We included 164 cases of inoperable locally advanced ESCC (II‐III stage, UICC2002) diagnosed from June 2009 to June 2014. The tumor tissue samples were collected by esophageal endoscopy. All the patients received concurrent chemoradiotherapy (CCRT). The gross tumor volume (GTV) was defined as the primary lesion determined by esophageal endoscopy and CT scan. The gross tumor volume of lymph nodes (GTVnd) was defined as the metastatic lymph nodes determined by CT scan. The clinical target volume (CTV) for 60 Gy irradiation included GTV plus a 3 cm craniocaudal margin and GTVnd plus a 1 cm margin. The planning target volume (PTV) was defined as CTV plus 0.5 cm margins for uncertainty. The radiation dose of PTV ranged from 60.4 Gy to 70.0 Gy and the median dose was 65.0 Gy. The number of fractions ranged from 30 to 38 and the median number was 35. Elective nodal irradiation (40 Gy) of mediastinal and perigastric lymph nodes for all cases, cervical lymph nodes for an upper thoracic primary tumor, and celiac lymph nodes for a lower thoracic primary tumor was also performed. The radiation technique was 3D conformal therapy. Chemotherapy compromised two courses of infusion of cisplatin (75–100 mg/m^2^) on Days 1 and 29, and infusion of 5‐Fu (750–1000 mg/m^2^) on Days 1–4 and 29–32. This regimen was repeated every five weeks. This study was approved by the Institutional Review Board of Shandong Cancer Hospital (number of approval document: 20161005) and conformed to the Declaration of Helsinki. The outcome of the study will not affect the future management of the patients and their personal data has been secured.

### Clinical data and follow‐up

The clinical characteristics including age, sex, PS score, smoking history, location of primary tumor and clinical stage were collected from the medical records of the patients. Treatment response was determined by the Response Evaluation Criteria in Solid Tumors. Progression‐free survival (PFS) was defined as the time between the start of active treatment and date of disease progression. Overall survival (OS) was defined as the time between the start of active treatment and date of death.

### Immunohistochemistry staining (IHC)

The ESCC tumor tissues from paraffin embedded blocks were cut into 4 μm sections, deparaffinized and rehydrated by xylene and graded alcohol. Heat‐induced antigen retrieval was achieved under high pressure for 20 minutes at 100°C using Bond Epitope Retrieval Solution 1 (Leica Microsystems). The sections were soaked in 3% hydrogen peroxide solution for 10 minutes and incubated with 5% goat serum at room temperature for 30 minutes. The sections were incubated with primary antibodies against Nrf2 (Proteintech, USA, 16396‐1‐AP) and phosphorylated p62 (Ser351, MBL, Japan, M217‐3) at 4°C overnight. Biotin‐labeled second antibody was applied for 30 minutes, and the slides were then incubated with polymeric horseradish peroxidase immunoglobulin G reagent for 30 minutes. DAB regent kit (ZSGB‐BIO, China) was used to detect antigen‐antibody binding. Finally, hematoxylin was applied for five minutes to counterstain the nuclei.

Each slide was independently evaluated by two pathologists and the conflicting results were resolved by using a multi‐headed microscope. The expression of p‐p62 and nuclear Nrf2 were quantified by the quick score (Q score), which was determined by multiplying the percentage of positive cells (0–100%) by intensity (0, 1, 2 or 3).^32^ The cutoff value to clarify “negative or low expression” and “positive or high expression” was the median value of Q scores. Patients with Q scores higher than or equal with the median value were classified as “positive or high expression.” Patients with Q scores lower than the median value were classified as “negative or low expression.”

### Statistical analysis

The association of p‐p62 and nuclear Nrf2 with clinical characteristics and treatment response was analyzed by χ^2^ test. Spearman test was used to determine the correlation between expression of p‐p62 and nuclear Nrf2. PFS and OS curves were constructed by the Kaplan‐Meier method, and differences in survival were compared by using the log‐rank test for univariate analysis. The factors with *P*‐value lower than 0.1 were included in the Cox proportion regression models. All two‐sided *P*‐values <0.05 were considered statistically significant. Statistical analysis was performed using SPSS version 22.0.

## Results

### Clinical characteristics and expression of p‐p62 and nuclear Nrf2

**Figure 2:**
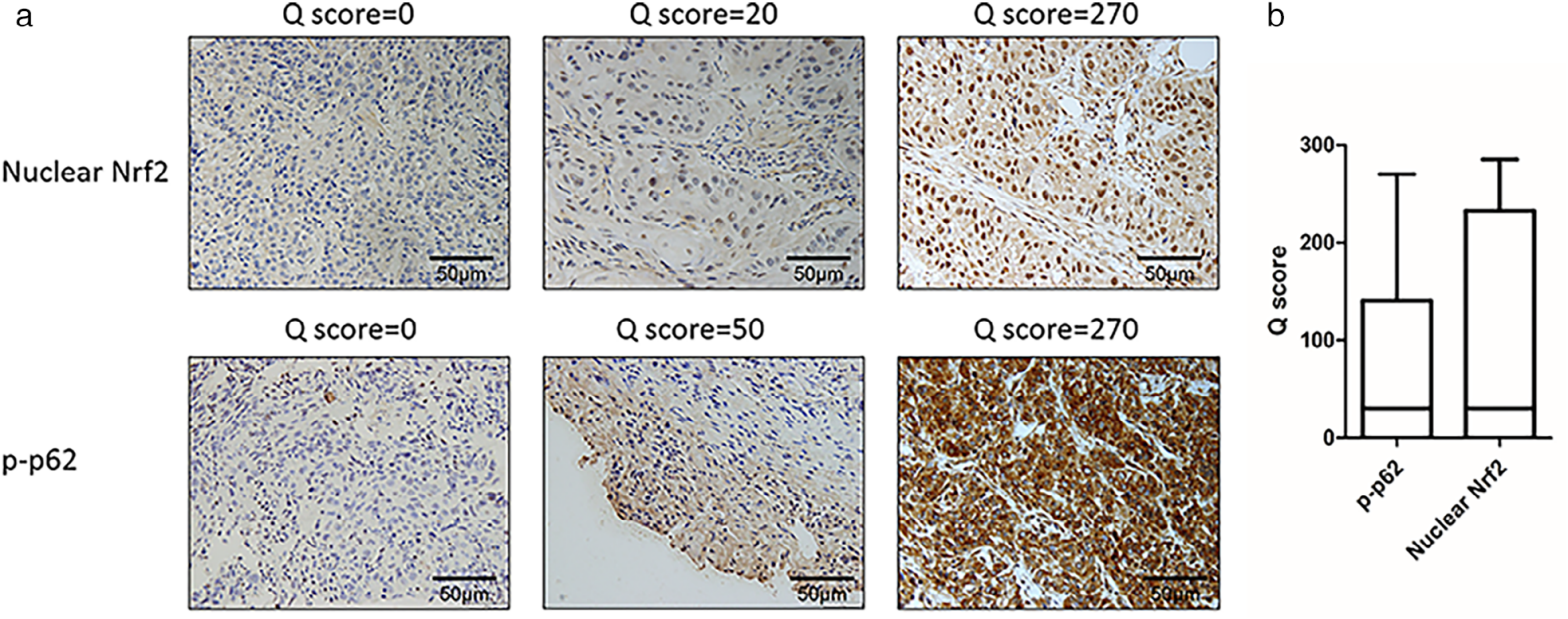
Representative IHC staining of p‐p62 and nuclear Nrf2. (**a**) Representative cases of p‐p62 and nuclear Nrf2 staining (original magnification = 400, scale bar 50 μm). (**b**) The distribution of Q scores of p‐p62 and nuclear Nrf2 in ESCC tumor tissues. The median lines of boxes show the median values, the top and bottom lines of boxes represent the 75th and 25th percentiles, respectively; and the ends of whiskers represent the 10th and 90th percentiles.

This study included 164 cases of ESCC whose clinical characteristics are presented in Table 1. Several representative IHC staining results of p‐p62 and nuclear Nrf2 shown in Fig 2a,b exhibited the distribution of Q scores of p‐p62 and nuclear Nrf2. The expression levels of p‐p62 and nuclear Nrf2 were fluctuant among patients. The median Q scores of p‐p62 and nuclear Nrf2 were both 20. A total of70 cases (42.7%) showed low p‐p62 expression [p‐p62(−)] and 94 cases (57.3%) showed high p‐p62 expression [p‐p62(+)]. In addition, the number of cases with low Nrf2 expression [Nrf2(−)] and high Nrf2 expression [Nrf2(+)] was 73(44.5%) and 91(55.5%) respectively.

**Table 1:** The clinical characteristics of patients included in the study

Clinical characteristics	ESCC (*N* = 164)
Age	
Range (Median)	65(37–86)
Sex	
Male	119(72.6%)
Female	45(27.4%)
PS score	
0	52(31.7%)
1	88(53.7%)
2	24(14.6%)
Smoking history	
No	98(59.8%)
Yes	66(40.2%)
Location of primary tumor	
Cervical	19(11.6%)
Upper thoracic	50(30.5%)
Middle thoracic	60(36.6%)
Lower thoracic	35(21.3%)
cTNM stage	
IIA	34(20.7%)
IIB	12(7.3%)
III	118(72.0%)

### Relationship between p‐p62 or nuclear Nrf2 expression and clinical characteristics of ESCC

The relationship between p‐p62 or nuclear Nrf2 expression and clinical characteristics of ESCC are summarized in Table 2. The results showed that there was no significant relationship between the expression level of p‐p62 and clinical characteristics including age, sex, PS score, smoking history, location of primary tumor and clinical stage. The results of nuclear Nrf2 were similar.

**Table 2:** The relationship between p‐p62 and nuclear Nrf2 expression and clinical characteristics of ESCC

		p‐p62			Nuclear Nrf2		
	N	(−)	(+)	*P*‐value	(−)	(+)	*P*‐value
Total	164	70	94		73	91	
Age				0.889			0.957
<65	76	32	44		34	42	
≥65	88	38	50		39	49	
Sex				0.526			0.488
Male	119	49	70		51	68	
Female	45	21	24		22	23	
PS score				0.341			0.335
0	52	25	27		26	26	
1–2	112	45	67		47	65	
Smoking history				0.706			0.161
No	98	43	55		48	50	
Yes	66	27	39		25	41	
Location of primary tumor				0.110			0.253
Cervical	19	7	12		8	11	
Upper thoracic	50	17	33		26	24	
Middle thoracic	60	25	35		21	39	
Lower thoracic	35	21	14		18	17	
T stage				0.314			0.859
T2	21	12	9		9	12	
T3	118	49	69		54	64	
T4	25	9	16		10	15	
N stage				0.717			0.824
N0	35	14	21		15	20	
N1	129	56	73		58	71	
cTNM stage				0.928			0.773
IIA	34	14	20		15	19	
IIB	12	6	6		4	8	
III	118	50	68		54	64	

### Relationship between p‐p62 or nuclear Nrf2 expression and clinical response to CCRT

**Figure 3:**
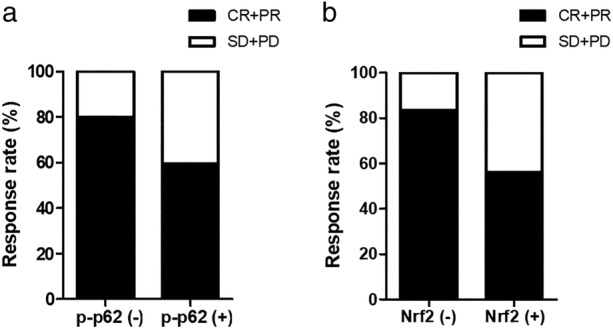
The relationship between p‐p62 or nuclear Nrf2 expression and clinical response to CCRT. (**a**) Treatment response of ESCC patients with low p‐p62 expression or high expression. Patients with high p‐p62 expression showed lower response rate to CCRT (*P* = 0.005). (**b**) Treatment response of ESCC patients with low nuclear Nrf2 expression or high expression. Patients with high nuclear Nrf2 expression showed lower response rate to CCRT (*P* < 0.001). CR, complete response; PR, partial response; SD, stable disease; PD, progressive disease.

All the 164 patients in our study received CCRT as initial treatment. A total of 112 patients (68.3%) achieved a complete response (CR) or partial response (PR), while 52 patients (31.7%) experienced stable disease (SD) or progressive disease (PD) (Fig 3a,b). Compared to patients with low p‐p62 expression, patients with high p‐p62 expression were associated with a poorer response (80.0% vs. 59.6%, *P* = 0.005). Similarly, patients with high nuclear Nrf2 expression were associated with a poorer response compared to patients with low expression (83.6% vs. 56.0%, *P* < 0.001) (Table 3).

**Table 3:** The relationship between p‐p62 or nuclear Nrf2 expression and clinical response to CCRT

		Treatment response		
		CR + PR	SD + PD	*P*‐value
p‐p62	Low expression (%)	56 (80.0%)	14 (20.0%)	0.005
	High expression (%)	56 (59.6%)	38 (40.4%)	
Nuclear Nrf2	Low expression (%)	61 (83.6%)	12 (16.4%)	<0.001
	High expression (%)	51 (56.0%)	40 (44.0%)	
Total		112 (68.3%)	52 (31.7%)	

### Correlation between expression of p‐p62 and nuclear Nrf2 in ESCC

**Figure 4:**
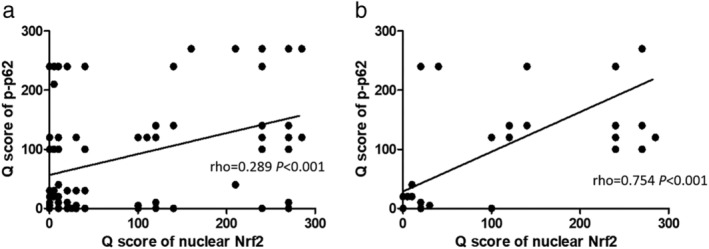
The correlation between expression of p‐p62 and nuclear Nrf2 in ESCC. (**a**) Among all the ESCC patients (*N* = 164), the expression of p‐p62 was positively correlated with the expression of nuclear Nrf2, rho = 0.289, *P* < 0.001. (**b**) Among patients with no response to CCRT (*N* = 52), the expression of p‐p62 was positively correlated with the expression of nuclear Nrf2, rho = 0.754, *P* < 0.001.

The expression of p‐p62 was positively correlated with nuclear Nrf2 and the rho value was 0.289 (Fig 4a) in all 164 patients. When the correlation between expression of p‐p62 and nuclear Nrf2 only was analyzed among patients with no response to CCRT, the rho value was 0.754 (Fig 4b).

### Association between p‐p62 or nuclear Nrf2 expression and prognosis after CCRT

**Figure 5:**
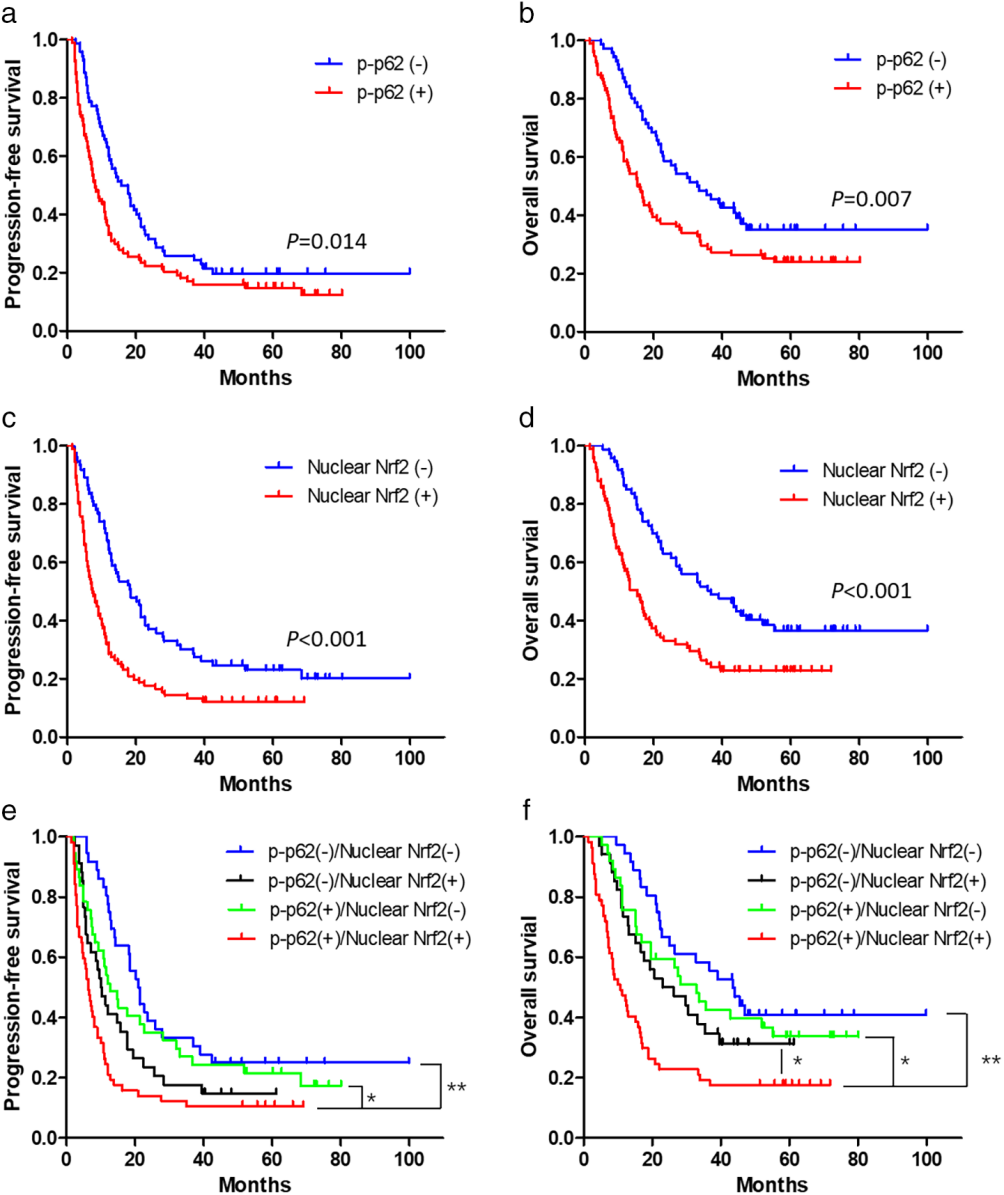
The PFS and OS curves of ESCC patients classified by expression of p‐p62 and nuclear Nrf2. (**a**) The PFS curves of ESCC patients classified by the expression of p‐p62. Patients with high p‐p62 expression had significantly shorter PFS. Median PFS: 15.7 months versus 8.0 months, *P* = 0.014. (**b**) The OS curves of ESCC patients classified by the expression of p‐p62. Patients with high p‐p62 expression had significantly shorter OS. Median OS: 32.7 months versus 15.3 months, *P* = 0.007. (**c**) The PFS curves of ESCC patients classified by the expression of nuclear Nrf2. Patients with high nuclear Nrf2 expression had significantly shorter PFS. Median PFS: 18.5 months *vs*. 7.4 months, *P* < 0.001. (**d**) The OS curves of ESCC patients classified by the expression of nuclear Nrf2. Patients with high nuclear Nrf2 expression had significantly shorter OS. Median OS: 36.7 months versus. 15.2 months, *P* < 0.001. (**e**) The PFS curves of ESCC patients classified by expression of p‐p62 and nuclear Nrf2. The median PFS of patients in p‐p62(−)/nuclear Nrf2(−), p‐p62(−)/nuclear Nrf2(+), p‐p62(+)/nuclear Nrf2(−) and p‐p62(+)/nuclear Nrf2(+) group was 21.0 months, 10.0 months, 12.7 months and 6.4 months respectively. *P < 0.05, **P < 0.001. (**f**) The OS curves of ESCC patients classified by expression of p‐p62 and nuclear Nrf2. The median OS of patients in p‐p62(−)/nuclear Nrf2(−), p‐p62(−)/nuclear Nrf2(+), p‐p62(+)/nuclear Nrf2(−) and p‐p62(+)/nuclear Nrf2(+) group was 43.4 months, 23.0 months, 32.8 months and 10.7 months respectively. *P < 0.05, **P < 0.001.

The PFS and OS curves classified by expression levels of p‐p62 or nuclear Nrf2 are summarized in Fig 5a‐d. The results of log‐rank test revealed that high p‐p62 expression was significantly associated with poorer PFS and OS. Similarly, high nuclear Nrf2 expression was also associated with poorer long‐term survival.

We subsequently divided patients into four groups as p‐p62(−)/nuclear Nrf2(−), p‐p62(−)/nuclear Nrf2(+), p‐p62(+)/nuclear Nrf2(−) and p‐p62(+)/nuclear Nrf2(+) group. The PFS and OS curves of the four groups are presented in Fig 5e,f. The PFS of patients in p‐p62(+)/nuclear Nrf2(+) group was significantly shorter than those in p‐p62(−)/nuclear Nrf2(−) or p‐p62(+)/nuclear Nrf2(−) group, but was not significantly different from patients in p‐p62(−)/nuclear Nrf2(+) group. Furthermore, the OS of patients in p‐p62(+)/nuclear Nrf2(+) group was significantly shorter than patients in the other three groups.

The results of univariate survival analysis revealed that PS score, N stage and treatment response were significantly associated with PFS besides expression of p‐p62 and nuclear Nrf2 (Table 4). The factors significantly associated with OS were sex, PS score, location of primary tumor, treatment response and expression of p‐p62 and nuclear Nrf2 (Table 5). Furthermore, the results of multivariate survival analysis showed that treatment response and expression of nuclear Nrf2 were independent prognostic factors for PFS. The independent prognostic factors for OS were sex, treatment response and expression of p‐p62 and nuclear Nrf2. In conclusion, the expression of p‐p62 and nuclear Nrf2 were critical factors for long‐term prognosis of ESCC patients after CCRT.

**Table 4:** The univariate and multivariate analysis of PFS

			Multivariate analysis	
Clinicopathological characteristics		Univariate analysis*P*‐value	HR (95% CI)	*P*‐value
Age	<65 vs. ≥65	0.066	0.793 (0.555–1.133)	0.202
Sex	Female vs. Male	0.966	‐	‐
PS score	0 vs. 1–2	0.01	0.840 (0.557–1.266)	0.404
Smoking history	No vs. Yes	0.847	‐	‐
Location of primary tumor	Cervical/upper thoracic vs. middle thoracic/lower thoracic	0.489	‐	‐
T stage	T2/3 vs. T4	0.54	‐	‐
N stage	N0 vs. N1	0.014	0.636 (0.403–1.002)	0.062
Treatment response	CR/PR vs. SD/PD	<0.001	0.355 (0.233–0.541)	<0.001
Nuclear Nrf2	Low vs. High	<0.001	0.598 (0.411–0.870)	0.007
p‐p62	Low vs. High	0.014	0.780 (0.545–1.116)	0.174

**Table 5:** The univariate and multivariate analysis of OS

			Multivariate analysis	
Clinicopathological characteristics		Univariate analysis*P*‐value	HR (95% CI)	*P*‐value
Age	<65 vs. ≥65	0.821	‐	‐
Sex	Female vs. Male	0.027	0.558 (0.356–0.874)	0.011
PS score	0 vs. 1–2	0.04	0.758 (0.485–1.186)	0.225
Smoking history	No vs. Yes	0.119	‐	‐
Location of primary tumor	Cervical/upper thoracic vs. middle thoracic/lower thoracic	0.028	0.669 (0.447–1.002)	0.06
T stage	T2/3 vs. T4	0.542	‐	‐
N stage	N0 vs. N1	0.519	‐	‐
Treatment response	CR/PR vs. SD/PD	<0.001	0.412 (0.269–0.632)	<0.001
Nuclear Nrf2	Low vs. High	<0.001	0.623 (0.421–0.922)	0.018
p‐p62	Low vs. High	0.007	0.626 (0.422–0.929)	0.02

## Discussion

In this study, we clarified the expression of p‐p62 and nuclear Nrf2 in ESCC and their association with patients' treatment response and long‐term prognosis after CCRT. Nrf2 is an important transcription factor and could only bind with ARE in the nucleus, so we focused on the expression of nuclear Nrf2 in our study but not the total expression of Nrf2 as in previous studies. The results of IHC revealed that the expression levels of p‐p62 and nuclear Nrf2 among patients were fluctuant, which implied the interindividual heterogeneity of tumors. Interestingly, although the expression of p‐p62 and nuclear Nrf2 were not stable in ESCC tumor tissues, there was a significantly positive correlation between the expression of p‐p62 and nuclear Nrf2, which was in accordance with the results of previous studies. In a homeostatic state, Keap1 combines with Nrf2 in the cytoplasm, which leads to the degradation of Nrf2 and keeps the expression of Nrf2 at low level.^15^, ^16^ However, mTORC1 phosphorylates p62 at Ser349 under stressed conditions, and p‐p62 competitively combines with Keap1, which induces the release and activation of Nrf2.^19^

More importantly, the positive correlation between expression of p‐p62 and nuclear Nrf2 was more obvious in ESCC patients showing no response to CCRT. This indicates that the relationship between p‐p62 and nuclear Nrf2 could affect the response of ESCC patients to CCRT. Further investigation showed that patients with high expression of p‐p62 or nuclear Nrf2 had a lower response rate to radiotherapy. We speculate that this is related to the role of ROS in the radioresistance and the function of target genes of Nrf2. ROS induced by radiotherapy could lead to the damage of DNA^4^ and the oxidation of biomacromolecules such as protein and lipid,^5^, ^6^ which mediates the anti‐tumor effects of radiotherapy. Recent studies have revealed that the aberrant clearance of ROS is one of the mechanisms of radioresistance of ESCC.^12^, ^13^, ^14^ In addition, the anti‐tumor effects of chemotherapy regimens used in CCRT of ESCC, cisplatin and 5‐fluorouracil, partially depend on ROS.^33^, ^34^, ^35^ As an important transcription factor, the target genes of Nrf2 include those encoding multiple antioxidative proteins such as NAD(P)H quinone oxidoreductase 1 (NQO1), glutathione S‐transferases (GSTs) and heme oxygenase‐1 (HMOX1).^36^, ^37^ The excessive activation of Nrf2 could eliminate ROS by these proteins, which mediates the radioresistance and anticancer agent tolerance. Previous studies have demonstrated that high expression of Nrf2 is involved in the mechanisms of radioresistance of esophageal cancer, breast cancer, prostate cancer and lung cancer.^25^, ^26^, ^27^, ^28^, ^29^, ^30^, ^31^ The study by Kawasaki *et al*.^30^ proved that the expression level of Nrf2 could predict the treatment response and long‐term prognosis of ESCC patients after CCRT. But the cohort sample in this study was relatively small. In addition, this study analyzed the total expression level of Nrf2 but not the nuclear Nrf2. Zhang *et al*.^28^ analyzed the impacts of expression of the nuclear Nrf2, cytoplasmic Nrf2 and Keap1 on treatment response to CCRT in ESCC patients. The results showed that only expression of nuclear Nrf2 had prognostic value, while the expression of cytoplasmic Nrf2 and Keap1 showed no significance. Previous studies related to aberrant activation of Nrf2 mainly focused on the mutation of the *NFE2L2* gene, especially in ESCC. The mutation rate of *NFE2L2* gene is 5% to 20% in ESCC. The mutant loci often locate in the regions affecting the binding of Nrf2 to Keap1, which means the mutation of *NFE2L2* gene usually decreases the binding affinity between Nrf2 and Keap1 and leads to the constant activation of Nrf2.^29^, ^31^, ^38^ In our study, the tumor tissue samples were collected by esophageal endoscopy and the gene sequencing was not applied due to the limited volume of tissue. However, the positive rate of nuclear Nrf2 was relatively high in our study, which suggests that there are other mechanisms mediating the abnormal activation of Nrf2 in ESCC. Combined with our results, the regulation of nuclear Nrf2 by p‐p62 might be the leading mechanism in radioresistance of ESCC.

The univariate analysis revealed that high expression of p‐p62 or nuclear Nrf2 was associated with shorter OS and PFS, especially when both the expression of p‐p62 and nuclear Nrf2 were high. Multivariate analysis also showed that expression of p‐p62 and nuclear Nrf2 were independently associated with long‐term survival. The prognostic value of Nrf2 in ESCC was in accordance with the results of previous study.^28^, ^30^ Meanwhile, the role of p‐p62 in ESCC remains unclear. However, the excessive expression of p62 is observed in multiple solid carcinomas such as hepatocellular carcinoma, prostate cancer, lung cancer and head and neck squamous carcinoma. Moreover, the high expression of p62 indicates a poor prognosis.^21^, ^22^, ^39^, ^40^, ^41^ Therapy targeted *p62* gene inhibit the proliferation of hepatocellular carcinoma in vivo and in vitro.^19^ The over aggregation of p62 is considered as one of reasons for renal cell carcinoma.^42^ In addition, the elevated expression of p62 induced by constant activation of K‐Ras leads to pancreatic ductal adenocarcinoma.^43^ In contrast, knockdown of *p62* gene inhibits pulmonary adenocarcinoma induced by *Ras*.^44^ More importantly, recent studies have proved that besides the phosphorylation of p62 by mTORC1, the aggregation of p62 also leads to its self‐phosphorylation at S349 and constant activation of Nrf2.^19^ This promotes the growth of tumors^19^ and leads to antitumor agent tolerance.^24^ We speculate that blockage of p62‐Keap1‐Nrf2 pathway could not only increase the radiosensitivity of ESCC, but also bring long‐term benefits.

There are several limitations in our study. A previous study has proved that pretreatment body mass index (BMI) was an independent prognostic factor for long‐term survival in esophageal cancer patients and BMI loss showed impact on OS.^45^ However, the relevant data were deficient in our study and other potential confounders, such as state of nutrition, were not included.

In conclusion, the expression levels of p‐p62 and nuclear Nrf2 are fluctuant among ESCC patients. The expression of p‐p62 is positively correlated with nuclear Nrf2, especially in patients with no response to CCRT. The high expression of p‐p62 and nuclear Nrf2 are associated with radioresistance and poor prognosis in ESCC. The p62‐Keap1‐Nrf2 pathway might be a new target for the radiosensitization of ESCC.

## Disclosure

The authors declare that there is no conflict of interest.
